# A Joint-Level Hybrid Framework for Gait Analysis Using Camera–IMU Fusion and LSTM-Based Temporal Correction

**DOI:** 10.3390/s26123828

**Published:** 2026-06-16

**Authors:** Eunju Ha, Jong-Wook Kim

**Affiliations:** Department of Electronic Engineering, Seunghak Campus, Dong-A University, Busan 49315, Republic of Korea; 19731jmj@naver.com

**Keywords:** gait analysis, sensor fusion, joint angle estimation, inertial measurement unit, RGB camera, LSTM

## Abstract

Gait analysis is an essential tool in clinical domains for diagnosing musculoskeletal disorders and evaluating rehabilitation, yet traditional marker-based systems are limited by high costs and spatial constraints. To overcome these challenges, this study proposes and evaluates a joint-level hybrid framework that integrates a single RGB camera with two shoe-mounted inertial measurement units (IMUs) to leverage their complementary strengths. The camera-based module estimates hip and knee sagittal joint angles using 3D pose estimation, where the DEAS optimization algorithm aligns estimated coordinates with a humanoid model, and an LSTM-based refinement network corrects hip angles by referencing more accurately estimated knee data. Simultaneously, the IMU-based module estimates sagittal ankle angles through kinematic chain relationships that combine camera-derived proximal joint information with IMU-measured foot orientation. Experimental validation with 11 healthy participants in a controlled laboratory environment demonstrates promising estimation performance, achieving an average mean absolute error (MAE) of 7.89° and RMSE of 10.09° on the held-out test set across sagittal hip, knee, and ankle angles. Leave-one-subject-out (LOSO) cross-validation of the LSTM correction model further confirmed its generalizability, yielding an average MAE of 6.40° across bilateral hip angles. By accurately mitigating the trunk-inclination-induced overestimation of hip angles with a minimal sensor configuration (one camera and two IMUs), the proposed framework provides a practical and interpretable approach for portable lower limb gait analysis.

## 1. Introduction

Gait analysis serves as an essential assessment tool in clinical and healthcare domains, including the diagnosis of musculoskeletal disorders, evaluation of rehabilitation treatment efficacy, and prediction of fall risk [1,2]. The kinematic information of lower limb joints quantitatively reflects the underlying causes of gait dysfunction, providing crucial evidence for treatment planning and rehabilitation protocol design [3,4]. Consequently, there has been increasing demand for practical gait analysis systems capable of repeated measurements in clinical settings and daily environments, with potential extension to long-term monitoring [5].

Traditionally, marker-based optical 3D motion capture systems (e.g., Vicon, Qualisys) have served as the gold standard for gait analysis [6,7]. While these systems provide high accuracy and reproducibility, their high cost, complex installation and operation requirements, and dedicated space constraints limit their routine clinical application. To overcome these limitations, markerless camera-based motion capture technologies have recently been applied to gait analysis, with active research investigating their validity and reliability [8,9,10].

Advances in computer vision have led to the development of 3D human pose estimation methods using single RGB cameras. Methods such as MediaPipe Pose (MPP) [11], Hybrid Analytical-Neural Inverse Kinematics (HybrIK) [12], and ViTPose [13] estimate 2D or 3D joint positions applicable to gait analysis. However, camera-based methods demonstrate relatively stable performance at proximal joints (hip and knee) but exhibit substantially increased estimation errors at distal joints (ankle) due to occlusion, rapid movement, and ground contact [10,14,15]. Despite ankle joint angles being essential for detecting gait events including heel strike and push-off, as well as for foot biomechanical analysis, achieving clinically acceptable accuracy with a single camera alone remains challenging.

Inertial measurement units (IMUs), comprising accelerometers and gyroscopes, measure segmental motion with high temporal resolution and have attracted attention as viable alternatives for real-world gait analysis [16,17]. Foot-mounted IMUs can directly observe foot motion during the gait cycle, enabling precise detection of key gait events such as heel strike and toe-off [16,17], and facilitating stable extraction of spatiotemporal gait parameters including walking speed, stride length, and step time [18]. Additionally, compact and lightweight sensors minimize wearing burden while enabling stable measurements during rapid movements and short walking distances. However, shoe-mounted IMUs can directly measure foot orientation but cannot directly observe ankle joint rotation, and reconstructing the complete lower limb kinematic chain requires multiple IMU placements [16,19]. Furthermore, accumulated drift over time and position estimation errors in the global coordinate system limit the ability of IMUs alone to reliably compute the kinematics of all lower limb joints.

Given this background, sensor fusion combining cameras and IMUs has been evaluated as an approach that can leverage the advantages of each sensor while mutually compensating for their limitations [19,20]. However, existing fusion studies have faced practical constraints for clinical application, requiring either multiple IMU sensors (five or more) with complex inter-sensor calibration [20,21], or end-to-end learning-based approaches demanding large-scale training data and high computational costs while reducing interpretability [22,23].

This study proposes and evaluates a joint-level hybrid framework centered on camera-based gait analysis, complementing the camera’s weak ankle joint estimation capabilities with IMU-based estimation. Rather than directly fusing raw sensor measurements, the proposed approach utilizes joint-level integration and kinematic joint relationships, leveraging the complementary strengths of the sensors by ensuring that each sensor primarily contributes to the joint it can most accurately observe. The system is validated as a preliminary feasibility study under controlled laboratory conditions with healthy participants.

The camera-based module computes joint angles for hip (sagittal and coronal planes) and knee (1 DoF, sagittal plane) through markerless 3D pose estimation. For joint angle estimation, we first use the DEAS [24] optimization algorithm to adjust the joint angles so that the 3D joint coordinates estimated from the camera align with those of the humanoid model. Then, using the knee joint angles—which were estimated with relatively higher accuracy—as a reference, we applied an LSTM to correct joint angles with lower accuracy, such as those of the sagittal and coronal hip [25]. The IMU-based module estimates the ankle angle (2 DoF: sagittal and coronal planes) and transversal hip joint angle through kinematic chain relationship using orientation information from shoe-mounted IMU sensors. This architecture ensures clinical applicability with minimal sensor configuration (one camera and two IMUs attached to both feet) while providing modularity for independent improvement of each module and high interpretability.

The main contributions of this study are as follows:

1. Joint-level modular fusion architecture: A hybrid framework that assigns each sensor modality (camera, IMU) to the anatomical joints and planes where it performs best, rather than performing black-box end-to-end fusion.
2. LSTM-based temporal correction: A dual-input LSTM network that leverages reliable knee angle sequences to refine camera-estimated hip joint angles, effectively compensating for systematic errors in monocular 3D pose estimation.
3. Kinematic chain-based ankle estimation: A method for estimating sagittal ankle angles by combining camera-derived proximal joint information with IMU-measured foot orientation through biomechanical kinematic chain relationships.
4. Validation with minimal sensor configuration: Experimental evaluation using only a single RGB camera and bilateral shoe-mounted IMUs, validated against a Vicon reference system with LOSO cross-validation across 11 participants.

The remainder of this paper is organized as follows. Section 2 reviews related work in camera-based, IMU-based, and fusion approaches for gait analysis. Section 3 describes the proposed joint-level hybrid framework, including the camera-based and IMU-based modules, their integration strategy, the experimental setup, participants, and evaluation methods. Section 4 presents the experimental results, including ablation analysis and LOSO cross-validation. Finally, Section 5 discusses the findings and concludes with future research directions.

## 2. Related Work

Table 1 summarizes representative studies in camera-based, IMU-based, and camera–IMU fusion approaches for lower limb gait analysis, highlighting their sensor configurations, target joints, and main limitations.

### 2.1. Camera-Based Markerless Gait Analysis

Advances in deep learning-based human pose estimation have enabled markerless gait analysis using monocular RGB cameras as a practical alternative to marker-based optical systems [8,9,10]. Methods such as OpenPose [31], MediaPipe Pose [11], HybrIK [12], and ViTPose [13] estimate 2D or 3D joint positions applicable to clinical gait assessment.

Validation studies have consistently reported a proximal-to-distal gradient in estimation accuracy. Kanko et al. [9] conducted a concurrent assessment of marker-based and markerless motion capture for gait kinematics, demonstrating acceptable accuracy at the hip and knee joints but not systematically evaluating ankle performance. Needham et al. [10] compared several 3D pose estimation methods and confirmed that ankle estimation errors are substantially larger than those at proximal joints due to occlusion, rapid foot motion, and depth ambiguity. Stenum et al. [15] demonstrated reliable spatiotemporal gait parameters from two-dimensional video-based analysis but noted limited accuracy for ankle kinematics. Kim et al. [26] proposed a humanoid model-based optimization using DEAS to improve joint angle estimation from MediaPipe Pose, achieving improved overall performance but acknowledging persistent ankle limitations. These findings indicate that camera-based methods alone cannot achieve clinically acceptable accuracy at the ankle joint, motivating the integration of complementary sensing modalities.

### 2.2. IMU-Based Gait Analysis

Inertial measurement units (IMUs) have been widely adopted for wearable gait analysis due to their portability and high temporal resolution [16,17,18]. Seel et al. [16] demonstrated that IMU-based ankle plantar/dorsiflexion measurement can achieve RMSE of approximately 1° using gyroscope integration with a complementary filter, but this requires sensors on both sides of each joint and provides no information on global body posture or segment position.

Huang et al. [23] demonstrated real-time pose reconstruction using six sparse IMUs; however, the task remains inherently ill-posed, as the lack of visual context introduces kinematic ambiguities that cannot be fully resolved by inertial data alone. Wang et al. [27] proposed a subject-independent TCN (Temporal Convolutional Network)-BiLSTM model using three IMUs to estimate lower limb joint angles across multiple locomotion activities, outperforming standalone LSTM and ANN models but still requiring multiple sensors per leg. Jocham et al. [28] validated highly accurate foot angle trajectory measurement using foot-mounted IMUs without magnetometer data, reporting RMSE below 1° for pitch and roll across both normal and pathological gait patterns. Xiang et al. [29] integrated an LSTM framework for predicting ankle joint biomechanics from foot-mounted inertial sensor data, demonstrating high prediction accuracy for ankle angles and torques but limited to single-joint estimation without kinematic chain reconstruction.

These studies demonstrate that IMUs excel at local orientation measurement, particularly at the foot, but reconstructing the complete lower limb kinematic chain from minimal sensor configurations remains challenging without complementary global pose information [19,23].

### 2.3. Camera–IMU Fusion Approaches

Camera–IMU fusion approaches leverage the complementary characteristics of each modality: cameras provide global spatial context while IMUs offer local orientation accuracy unaffected by occlusion [20,21,22]. Von Marcard et al. [20] combined six IMUs with a moving camera for accurate 3D pose recovery, but the approach requires complex multi-sensor calibration. Malleson et al. [21] presented real-time multi-person motion capture from multi-view video and IMUs, demonstrating the feasibility of simultaneous capture but requiring a multi-camera setup that limits clinical deployment. Gilbert et al. [22] fused multi-view video with 13 IMUs through an end-to-end deep learning pipeline, achieving high accuracy but at substantial computational cost with reduced interpretability.

Specifically for gait analysis, Yamamoto et al. [30] proposed fusing OpenPose with a single foot-mounted IMU to reduce ankle angle errors during walking. While their approach demonstrated that camera–IMU fusion could improve ankle estimation, it was limited to a single IMU configuration and did not incorporate temporal modeling or biomechanical constraints to ensure physiological validity throughout the gait cycle.

The present study addresses these limitations by proposing a joint-level modular framework that combines a single RGB camera with bilateral shoe-mounted IMUs (Table 1). Unlike end-to-end fusion approaches, the proposed method independently refines each sensor stream—applying DEAS optimization and LSTM-based temporal correction for camera-based hip and knee estimates, and kinematic chain relationship for sagittal ankle angle estimation by combining camera-derived proximal joint angles with IMU-measured foot pitch. Temporal alignment between the two sensor streams is achieved through normalized cross-correlation. This architecture maintains clinical interpretability while enabling independent improvement of each processing module.

## 3. Methodology

### 3.1. System Overview

**Figure 1:**
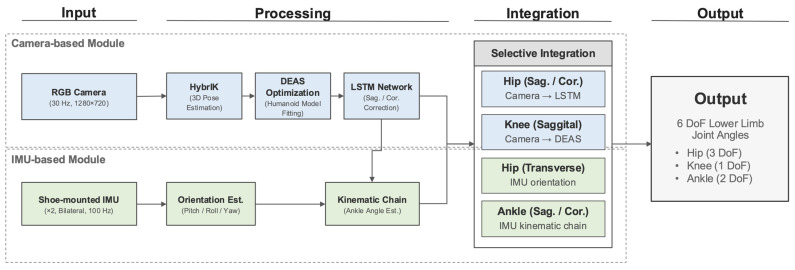
Overall structure of the proposed system.

The proposed gait analysis framework estimates lower limb joint angles using a single RGB camera and bilateral shoe-mounted IMU sensors (Figure 1). The system is based on a parallel processing architecture that considers the characteristics of each sensor modality, with selective integration performed at the joint level in the final stage.

The input data consists of two independent sensor streams. The camera stream comprises RGB images sampled at 30 Hz with a resolution of 1280 × 720 pixels. The IMU stream consists of triaxial acceleration and triaxial angular velocity signals collected at 100 Hz from sensors attached to the soles of both shoes. The two sensors are initially synchronized via a hardware trigger, with final temporal alignment achieved through cross-correlation-based synchronization described in Section 3.3.

The proposed method adopts a structure that processes each sensor stream independently rather than performing direct sensor-level fusion. In the camera-based stream, 3D joint positions are estimated using HybrIK and subsequently used to estimate biomechanical joint angles through humanoid model fitting by DEAS. The sagittal and coronal hip joint angles are then refined by applying an LSTM network using the knee joint angle as a reference input (Section 3.2). In the IMU-based approach, the pitch, yaw, and roll angles of both soles are initially estimated using IMU sensors mounted on both shoes. Then, the sagittal and coronal ankle joint angles are estimated from the kinematic chain relationships combining camera-derived proximal joint angles with IMU-measured foot orientation (Section 3.3). Through this joint-specific role assignment strategy, the proposed method effectively leverages the strengths of each sensor while compensating for the limitations of single-sensor approaches.

The monocular RGB camera is positioned behind the participant during walking. Although this posterior viewpoint does not directly observe the sagittal plane, HybrIK [12] estimates 3D joint positions from monocular images by employing a learned analytical-neural inverse kinematics approach that reconstructs full 3D body pose regardless of viewing angle. The DEAS optimization framework then fits a biomechanical humanoid model to these 3D positions, computing joint angles in all anatomical planes. The posterior placement was chosen to minimize occlusion of lower limb joints during forward walking, which is the primary limitation of frontal or lateral camera views in overground gait analysis.

### 3.2. Camera-Based Joint Angle Estimation

The camera-based module estimates 3D lower limb joint angles from monocular RGB images. The processing pipeline consists of two stages: (1) 3D pose estimation with humanoid model fitting, and (2) LSTM-based joint angle refinement.

#### 3.2.1. 3D Pose Estimation and Model Fitting

For an input RGB image sequence {$I_{t}$}, HybrIK [12] is applied to estimate 3D joint positions for the *t*-th frame, as summarized in Table 2. HybrIK was selected because it employs an analytical inverse kinematics approach based on twist-and-swing decomposition to recover 3D joint rotations from 2D keypoints, which structurally mitigates the depth ambiguity inherent in monocular images compared to purely regression-based methods. HybrIK combines 2D keypoint detection with inverse kinematics-based 3D reconstruction, and the output consists of joint positions $J_{t}\in \;R^{K\times 3}$ based on the SMPL body model, where K=24 denotes the total number of body joints. In this study, coordinates corresponding to the lower limb joints (hip, knee, and ankle) are extracted for subsequent processing.

However, the raw output of HybrIK represents 3D positions in the image coordinate system and cannot be directly converted to biomechanical joint angles. Furthermore, inter-frame consistency is not guaranteed, potentially resulting in temporal jitter and physiologically implausible poses. Our previous work [32] demonstrated that optimization-based model fitting using DEAS can effectively estimate joint angles from 2D pose estimation, but also revealed persistent estimation errors at distal joints, particularly the ankle. To address these issues, humanoid model-based optimization is performed. Rather than solving inverse kinematics analytically—which is intractable for a complex full-body humanoid model—we formulate the problem as a numerical optimization in which the joint angle parameters are directly searched to minimize the discrepancy between the AI-estimated and model-predicted joint positions.

The DEAS optimization framework simultaneously resolves 23 variables to fit the full-body humanoid model to HybrIK-estimated 3D joint coordinates. Although the optimization covers the entire body to ensure kinematic consistency, only the lower limb joint angles are extracted for the proposed gait analysis pipeline.

Model fitting is formulated as an optimization problem that minimizes the Mean Per Joint Position Error (MPJPE) between HybrIK-estimated positions and the forward kinematics output of the humanoid model:

(1) $$\theta^{*}=arg\;\underset{\theta }{min}\frac{1}{N_{J}}\sum_{j=1}^{N_{J}}{∥p_{j}^{HybrIK}-p_{j}^{FK}(\theta )∥}_{2}$$

where $N_{J}=6$ is the number of major joint coordinates used for optimization, comprising the bilateral hip, knee, and ankle joints. $p_{j}^{HybrIK}$ is the 3D position of the j-th joint estimated by HybrIK, and $p_{j}^{FK}(\theta )$ is the 3D position computed through forward kinematics using optimization variables θ. These variables comprise: (1) lower limb kinematics, including triaxial hip angles and sagittal knee flexion/extension; (2) global spatial parameters, such as a link-length scaling factor to compensate for distance-induced size changes and three camera-to-body relative orientations; and (3) trunk dynamics, represented by three lumbar spine joint angles. Following the optimization, the estimated hip and knee angles are extracted for subsequent processing stages.

The optimization is performed using DEAS [24], a derivative-free global optimization algorithm. DEAS encodes each real-valued variable as a binary string and combines bisectional search (BSS) with unidirectional search (UDS) for local search while employing a multistart strategy for global search. BSS refines the search resolution by appending a binary digit to the current string, whereas UDS traverses equal-resolution grid points along the promising direction detected by BSS. The algorithm adaptively adjusts the variable search order based on cost function sensitivity, achieving efficient convergence while minimizing MPJPE. The global convergence of DEAS has been proven within the generating set search framework [24]. Specifically, the optimization solves for 23 variables with 10 maximal binary encoding bits per variable and up to 10 restarts per frame, minimizing the MPJPE across six bilateral lower limb joints.

Due to the characteristics of monocular images, estimation accuracy varies across joints. The knee joint is clearly observable in images and exhibits relatively high accuracy. In contrast, the hip joint shows larger errors due to depth ambiguity and self-occlusion. To address this, an LSTM-based refinement network trained with Vicon ground truth is employed to correct the camera-estimated hip angles, as described in Section 3.2.2. The ankle region is particularly challenging, with low keypoint detection frequency and inter-frame position instability. Therefore, ankle joint angles are excluded from the DEAS optimization variables and are instead estimated using the IMU-based module described in Section 3.3.

#### 3.2.2. LSTM-Based Refinement Using Knee Reference

Although DEAS-based model fitting estimates hip and knee joint angles, hip accuracy in the sagittal plane remains lower than that of the knee. This is because knee flexion/extension is clearly observable in images, whereas the hip position is affected by depth ambiguity and self-occlusion. Additionally, ankle joint angles are excluded from DEAS optimization and are instead estimated through kinematic chain relationship using IMU-measured foot orientation, as described in Section 3.3.

To overcome these challenges, an LSTM-based refinement network is developed to rectify the DEAS-derived hip sagittal and coronal angles. By utilizing reliable knee joint sequences as a spatiotemporal reference, the network exploits the inherent coordination of human gait, treating the robust knee kinematics as an anchor to enhance the estimation accuracy of the hip joint [25].

**Figure 2:**
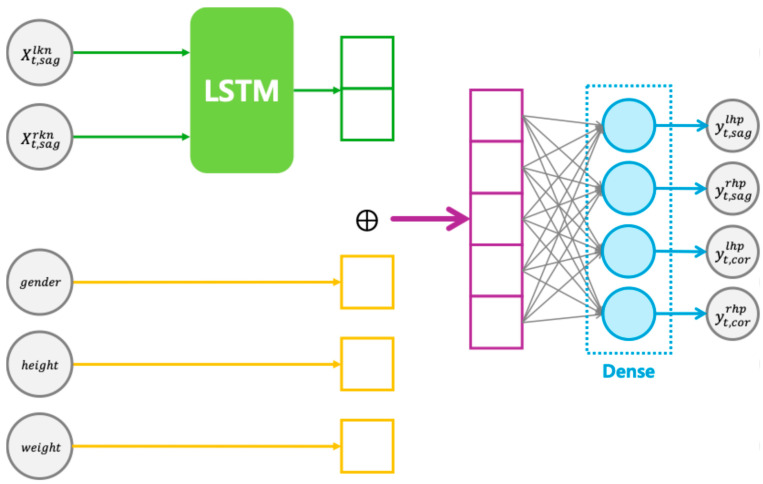
Input and output structure of the LSTM model.

The proposed LSTM network employs a dual-input architecture, as shown in Figure 2. The first input consists of bilateral sagittal knee angle sequences over a sliding window of 30 frames (window size = 30), corresponding to approximately 1 s at 30 Hz sampling rate, which is sufficient to capture the key phases of a typical gait cycle. The second input is a 3-dimensional participant attribute vector comprising sex, height, and weight. The LSTM layer contains 500 hidden units with dropout of 0.3 and recurrent dropout of 0.2. Features extracted from the LSTM are concatenated with the attribute vector and passed through a dense output layer with linear activation to produce a 4-dimensional output comprising bilateral hip sagittal and coronal angles. The loss function is defined as mean squared error (MSE):

(2) $$L_{LSTM}=\frac{1}{T}\sum_{t=1}^{T}{∥{\hat{y}}_{t}-y_{t}^{GT}∥}_{2}^{2}$$

where ${\hat{y}}_{t}\;$ denotes the predicted joint angles and $y_{t}^{GT}$ represents the ground truth from the Vicon system. Training was performed using the Adam optimizer with a learning rate of 0.001 for up to 200 epochs and a batch size of 32. Early stopping with a patience of 10 epochs was applied to prevent overfitting, and the learning rate was reduced by a factor of 0.2 when the validation loss plateaued for five consecutive epochs. All walking trials, including both overground and treadmill conditions, were used for model training to maximize the training data size given the limited number of participants. To evaluate generalizability, LOSO cross-validation was performed across all 11 participants, with 20% temporal validation split within each training fold for early stopping. During inference, knee joint angles estimated by DEAS are input to the LSTM to obtain refined hip joint angles. The LSTM outputs replace the DEAS estimates for hip sagittal and coronal plane angles, while the knee joint angles from DEAS are retained as the final output. The ankle sagittal and coronal angles are estimated through kinematic chain relationship using IMU-measured foot pitch, as described in Section 3.3.

### 3.3. Vision-Inertial Kinematic Fusion

This section presents the fusion framework for estimating the ankle sagittal angle by integrating the camera-derived lower limb kinematic chain with shoe-mounted IMU orientation measurements. The fusion process consists of two stages: (1) temporal synchronization between the two sensor streams and (2) estimation of sagittal and coronal ankles via kinematic chain relationship.

#### 3.3.1. IMU-Based Ankle Kinematics Estimation

The primary motivation for this fusion is the inherent instability of monocular RGB-based estimation in the distal lower extremities. Compared to the hip and knee, the ankle region is particularly susceptible to structural tracking errors due to high-velocity rotations during gait, complex foot–ground interactions, and abrupt directional changes during the toe-off phase.

To compensate for these limitations, we implement a dedicated IMU-based ankle estimation pipeline that operates in parallel with the HybrIK-based pathway used for hip and knee estimation. The raw triaxial accelerometer and gyroscope signals, sampled at 100 Hz, are first processed through a second-order Butterworth low-pass filter (cutoff frequency: 6 Hz) to eliminate high-frequency noise and subsequently downsampled to 30 Hz for temporal alignment with the vision data. The filtered signals are processed through a Madgwick complementary filter [33] (beta = 0.1) to estimate the absolute orientation (pitch, roll, yaw) of the foot sole. The filter orientation is reset at each detected heel-strike event to bound gyroscope drift accumulation within a single stride. The resulting orientation angles, combined with synchronized gait event information, are subsequently used in the kinematic chain relationship described in Section 3.3.2.

#### 3.3.2. Kinematic Chain Relationship

In our humanoid model, three body angles describe the relative orientation difference between the body coordinate frame and the camera-based coordinate frame: $\theta_{bd}$ (sagittal body angle) represents the forward/backward tilt of the body (positive for forward tilting), $\phi_{bd}$ (coronal body angle) represents the lateral tilt (positive for left tilting), and $\psi_{bd}$ (transversal body angle) represents the lateral rotation (positive for left rotation).

**Figure 3:**
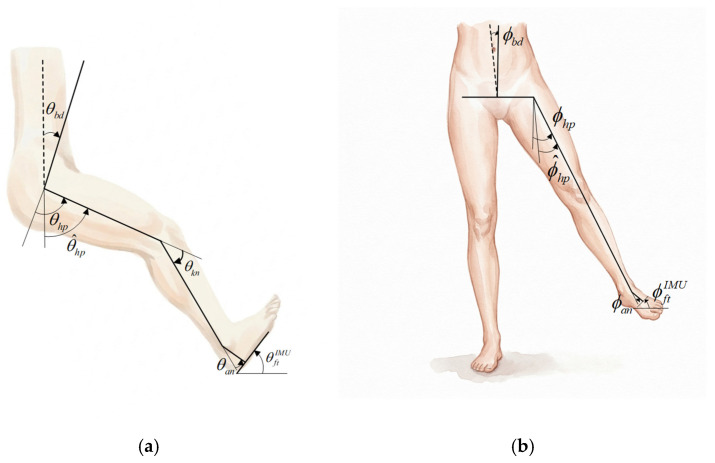
Definition of body angles ($\theta_{bd}$ and $\phi_{bd}$) and lower limb joint angles for gait analysis. The solid lines in the upper body represent the erroneous estimates extracted by HybrIK, while the dotted lines represent the actual upper body orientation. (**a**) Sagittal-plane parameters, including hip ($\theta_{hp}$) and knee ($\theta_{kn}$) angles. (**b**) Coronal plane parameters, including hip ($\phi_{hp}$) and ankle ($\phi_{an}$) angles. The symbols ${\hat{\theta }}_{hp}$ and ${\hat{\phi }}_{hp}$ represent the refined hip joint estimates, accounting for the fact that actual upper body angles are nearly zero during walking.

Figure 3 illustrates the geometric relationships among body angles and the lower limb joint angles when the human body is viewed in the sagittal and coronal planes. In Figure 3a, $\theta_{hp}$ characterizes the hip flexion angle in the sagittal plane, measured from the longitudinal axis of the sacrum—which serves as the vertical reference—to the thigh segment. In addition, $\theta_{kn}$ denotes the knee flexion angle, and $\theta_{an}$ denotes the ankle dorsiflexion angle. The angle $\theta_{ft}^{IMU}$ represents the pitch angle of the foot sole measured by the IMU sensor. In Figure 3b, $\phi_{hp}$ denotes the hip abduction angle measured from the vertical reference and $\phi_{an}$ denotes the ankle eversion angle. $\phi_{ft}^{IMU}$ represents the roll angle of the foot sole captured by the IMU sensor.

While these joint angles are derived from camera images via the DEAS process, the estimation accuracy is sometimes compromised. Specifically, although $\theta_{bd}$ and $\phi_{bd}$ are theoretically minimal during upright walking, the HybrIK model can produce inaccurate upper-body segments, resulting in the excessively high values shown in Figure 3. Due to the kinematic dependency on the trunk’s orientation within our model, errors in the estimated pelvic or sacral tilt inevitably result in a corresponding overestimation of $\theta_{hp}$ and $\phi_{hp}$, effectively distorting the derived hip flexion angles.

To mitigate the propagation of error from the upper body, the hip joint angles are recalibrated by subtracting the body tilt components ($\theta_{bd}$ and $\phi_{bd}$). Consequently, the refined hip flexion and abduction angles are expressed as ${\hat{\theta }}_{hp}$ and ${\hat{\phi }}_{hp}$, respectively, according to the following equation:

(3) $${\hat{\theta }}_{hp}=\theta_{hp}-\theta_{bd}$$

(4) $${\hat{\phi }}_{hp}=\phi_{hp}-\phi_{bd}$$

This correction reduces the influence of body angle estimation error and enables ${\hat{\theta }}_{hp}$ and ${\hat{\phi }}_{hp}$ to more accurately reflect the actual hip flexion and abduction angle.

Given the geometric constraints within the sagittal plane, the sagittal ankle joint angle can be derived from the following relationship:

(5) $$\theta_{ft}^{IMU}={\hat{\theta }}_{hp}-\theta_{kn}+\theta_{an}=\theta_{hp}-\theta_{bd}-\theta_{kn}+\theta_{an}$$

(6) $$\theta_{an}=\theta_{ft}^{IMU}-\theta_{hp}+\theta_{bd}+\theta_{kn}$$

In the same manner, the coronal ankle joint angle can be derived from the following relationship

(7) $$\phi_{ft}^{IMU}={\hat{\phi }}_{hp}+\phi_{an}=\phi_{hp}-\phi_{bd}+\phi_{an}$$

(8) $$\phi_{an}=\phi_{ft}^{IMU}-\phi_{hp}+\phi_{bd}$$

### 3.4. Participants and Protocol

All data were collected in our laboratory, as no publicly available datasets include synchronized monocular camera, bilateral shoe-mounted IMU, and Vicon reference data for the same walking trials. For this study, a total of 11 participants (seven males, four females; mean age 62.3 ± 13.8 years) were recruited. To capture diversity in gait patterns across age groups, the study included four adults (18–64 years) and seven older adults (65 years and above), as summarized in Table 3. All participants had no history of lower limb musculoskeletal or neurological disorders and were capable of independent ambulation without assistive devices. Written informed consent was obtained from all participants prior to data collection.

Each participant was assessed under two walking conditions. First, in the 10 m overground walking task, participants walked in a straight line on a level surface. Second, in the 60 s treadmill walking task, participants walked at a constant speed on a treadmill. Walking speed was set at three conditions: 80%, 100%, and 120% of each participant’s self-selected comfortable speed. For overground walking, a metronome was used to cue the gait cadence, and each speed condition was repeated twice. For treadmill walking, belt speed was directly adjusted, and each speed condition was performed once. A total of nine trials were conducted per participant (six overground walking trials + three treadmill walking trials). For treadmill walking, a 30 s familiarization period was provided before each measurement. A minimum rest period of 2 min was provided between trials to minimize fatigue effects.

### 3.5. Equipment

**Figure 4:**
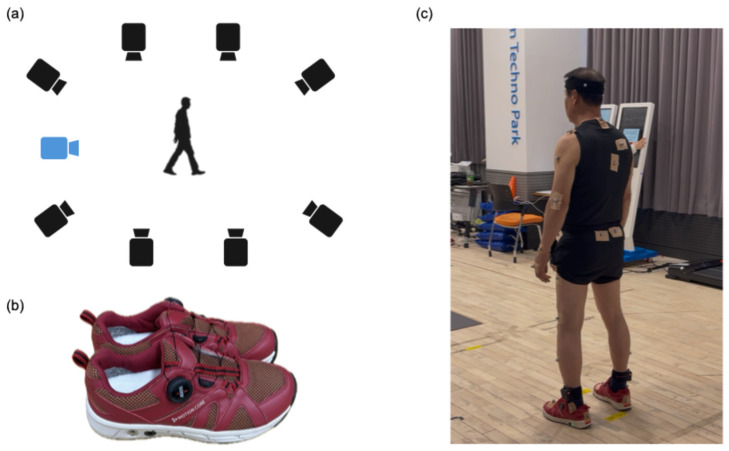
Experimental setup. (**a**) Measurement environment with Vicon infrared cameras (black) and a monocular RGB camera (blue), (**b**) shoe-mounted IMU sensors (Motioncore), and (**c**) participant during data acquisition with reflective markers and instrumented shoes.

Reference kinematic data were acquired using an eight-camera Vicon optical motion capture system operating at 100 Hz, as illustrated in Figure 4a,c. Thirty-nine reflective markers were attached to the participant’s body according to the Plug-in Gait marker set. Marker trajectories were processed using Vicon Nexus software (version 2.14; Vicon Motion Systems Ltd., Oxford, UK). To remove high-frequency noise, a fourth-order Butterworth low-pass filter with a cutoff frequency of 6 Hz was applied. The filtered trajectories were subsequently downsampled to 30 Hz to match the frame rate of the RGB camera.

As shown in Figure 4a, a monocular RGB-D camera (Intel RealSense D435i; Intel Corporation, Santa Clara, CA, USA) was mounted on a tripod positioned 2.3 m behind the participant at a height of 0.8 m. Only the RGB sensor was utilized, and video was recorded at a resolution of 1280 × 720 pixels at 30 frames per second. The depth sensor and built-in IMU were not used in this study. The recorded RGB video was used as input to HybrIK for frame-by-frame estimation of 3D joint positions and joint angles.

The shoe-mounted IMU-based gait analysis system shown in Figure 4b is Motioncore (JEIOS, Seoul, South Korea). The system comprises shoe-mounted data loggers and a data acquisition unit. The embedded IMU sensors measure triaxial acceleration (±16 g) and triaxial angular velocity (±2000°/s). The sensors are mounted on the soles of both shoes, and data were transmitted wirelessly via Bluetooth. Instrumented shoes were available in sizes ranging from 230 to 280 mm, and each participant wore an appropriately sized pair. The local sensor coordinate system was defined along the anteroposterior, mediolateral, and vertical axes. Data were sampled at 100 Hz, and bias calibration was performed in a stationary position prior to measurement. Temporal alignment across sensing modalities was performed during post-processing using a cross-correlation. Gait cycles were detected using heel-strike events identified from the vertical position (Z-coordinate) of heel markers for the Vicon reference system and ankle joint coordinates for the camera-based system.

### 3.6. Evaluation Metrics

The performance of the proposed method was evaluated using joint angles obtained from the optical motion capture system (Vicon) as the reference. Mean absolute error (MAE) and root mean square error (RMSE) were employed as quantitative evaluation metrics. All metrics were computed from time-aligned joint angle trajectories and were reported as mean ± standard deviation across all participants and repeated trials.

The MAE, representing the average absolute difference between estimated and reference joint angles, is defined as

(9) $$MAE=\frac{1}{N}\sum_{i=1}^{N}|\theta_{i}^{est}-\theta_{i}^{ref}|$$

where $\theta_{i}^{\mathrm{est}}$ and $\theta_{i}^{\mathrm{ref}}$ represent the estimated and reference joint angles at frame *i*, respectively, and *N* is the total number of frames.

The RMSE, which assigns greater weight to larger deviations, is defined as

(10) $$RMSE=\sqrt{\frac{1}{N}\sum_{i=1}^{N}{\left(\theta_{i}^{est}-\theta_{i}^{ref}\right)}^{2}}$$

Evaluation was conducted for lower limb joints, including hip, knee, and ankle joint angles in the sagittal plane. Left and right joints were evaluated separately, and the average performance across both sides was additionally reported.

## 4. Results

### 4.1. Joint Angle Trajectory Comparison

**Figure 5:**
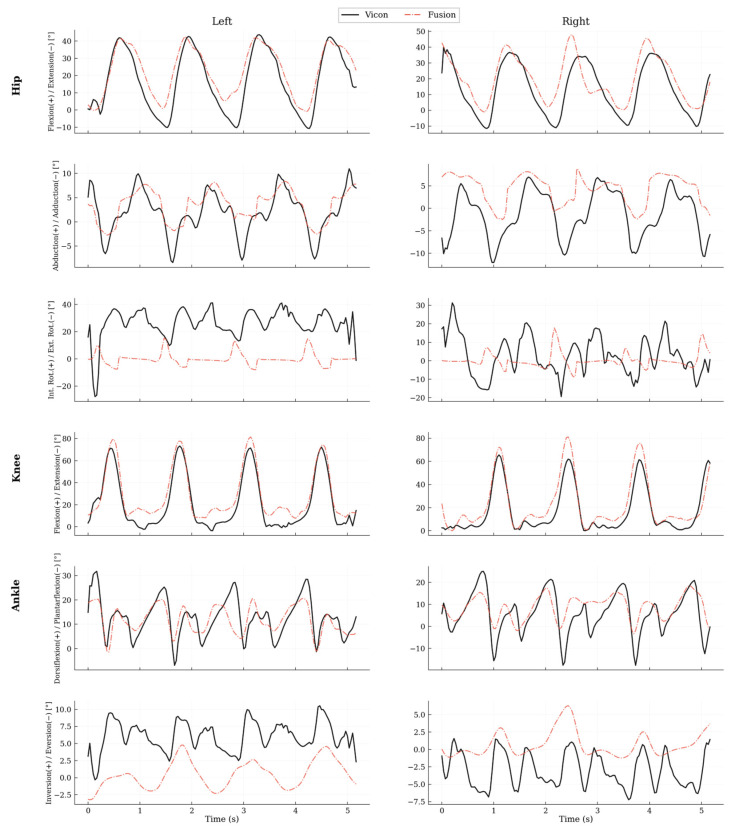
Comparison of lower limb joint angle trajectories between the Vicon system (black solid) and the proposed camera–IMU fusion method (red dash-dot).

Figure 5 compares the lower limb joint angle trajectories over a normalized gait cycle (0–100%) for the Vicon reference (black solid) and the proposed camera–IMU fusion method (red dash-dot). Solid lines and shaded regions denote the mean trajectories and ±1 standard deviation, respectively.

For the hip sagittal angle, the proposed method captured the overall flexion and extension pattern, though amplitude underestimation was observed during peak flexion due to monocular depth ambiguity. In the coronal plane, a complementary filter combining camera low-frequency trends with IMU roll high-frequency dynamics produced trajectories consistent with the reference. The transverse plane showed limited agreement, as absolute hip rotation could not be recovered from foot-mounted IMUs alone.

For the knee sagittal angle, the proposed method reproduced the overall flexion pattern during the swing phase, though the peak magnitude was underestimated compared to the reference.

For the ankle sagittal angle, the kinematic chain relationship fusing camera-derived proximal joint angles with IMU-measured foot pitch successfully captured the plantarflexion and dorsiflexion pattern. In the coronal plane, IMU roll with shank tilt compensation enabled estimation of inversion/eversion dynamics that the camera alone could not provide.

**Figure 6:**
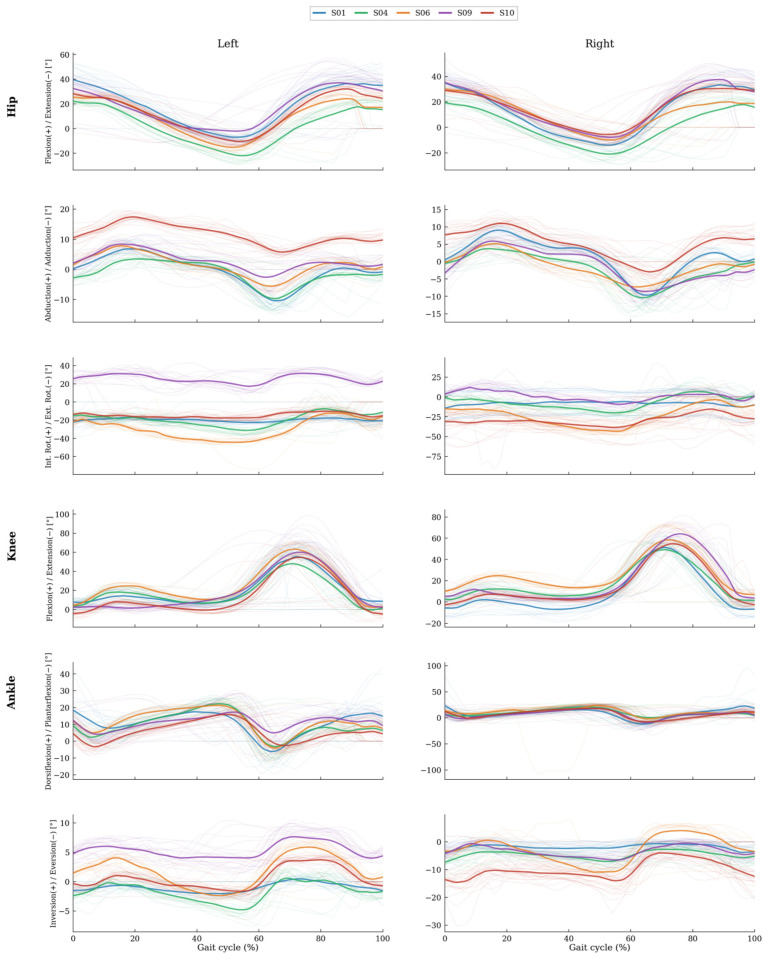
Gait cycle normalized lower limb joint angles obtained from a Vicon motion capture system for five selected subjects. Thin line: individual gait cycle; Thick line: average value per subject. S09 and S10 show high variability in the coronal and transverse planes.

Figure 6 presents gait cycle normalized joint angles from the Vicon system for five selected subjects. S09 and S10 exhibited notably higher variability in the coronal and transverse planes compared to the remaining subjects. This inter-subject variability in the reference measurements indicates that estimation errors should be interpreted in the context of inherent biomechanical differences across individuals, rather than solely as limitations of the proposed method.

As shown in Figure 6, while the hip, knee, and ankle joint angles in the sagittal plane demonstrate high intra-subject consistency, other joint angles exhibit substantial subject-specific offsets. This discrepancy is particularly pronounced in subjects S09 and S10, where the inconsistent offsets diminish the reliability of these measurements as a ground truth. Consequently, these non-sagittal components were omitted from the performance analysis to ensure a robust and fair evaluation of the proposed system’s accuracy.

### 4.2. Joint Angle Estimation Accuracy

Table 4 presents an ablation comparison of joint angle estimation errors on the held-out test set under two conditions: DEAS-only, where joint angles are estimated through DEAS optimization without LSTM correction, and the full proposed pipeline, which additionally incorporates LSTM-based hip refinement and IMU-based ankle estimation. Accuracy (100 − MAE/ROM × 100) is reported as a supplementary metric for comparison with prior work [25], noting that this metric is ROM-dependent and may not uniformly reflect clinical significance across joints.

The DEAS-only condition yielded an average MAE of 16.40° ± 4.03° and RMSE of 20.70° ± 4.66°, whereas the proposed pipeline reduced these to 7.89° ± 1.82° and 10.09° ± 2.08°, respectively—an overall MAE improvement of 8.51°. The improvement was most pronounced for knee sagittal angles (10.69–13.79°) and hip sagittal angles (9.65–11.69°), confirming that the LSTM-based temporal correction substantially enhances the estimation accuracy at joints affected by monocular depth ambiguity.

The proposed method achieved an overall average MAE of 7.89° and RMSE of 10.09° across all evaluated sagittal joints, with a mean accuracy of 85.5%. The ankle sagittal angle achieved the lowest MAE of 5.14–6.96°, confirming that the fusion of camera-derived proximal joint information with IMU-measured foot orientation effectively compensates for the camera’s limited accuracy at distal joints. The knee sagittal angle demonstrated MAE of 9.40–9.64° with accuracy of 87.0%; although the MAE is higher than the ankle, the high accuracy reflects the large ROM of knee flexion/extension, which is clearly observable from the posterior camera. Hip sagittal angles showed MAE of 7.31–8.87° after LSTM-based temporal refinement. A consistent left–right asymmetry was observed (R-Hip MAE approximately 1.6° higher than L-Hip), which may reflect a combination of inherent gait asymmetry across participants and depth estimation variability in monocular 3D pose reconstruction.

### 4.3. LOSO Cross-Validation

To evaluate the generalizability of the LSTM-based correction model across unseen individuals, leave-one-subject-out (LOSO) cross-validation was performed across all 11 participants. In each of the 11 folds, the LSTM was trained on data from 10 participants and evaluated on the held-out participant, ensuring complete subject independence between training and test sets. It should be noted that the LOSO evaluation uses Vicon reference knee angles as input to the LSTM and therefore represents the upper-bound performance of the correction model itself. The pipeline-level accuracy reported in Table 4, which includes camera-based estimation errors propagated through DEAS, reflects the actual end-to-end system performance.

The LOSO cross-validation yielded an overall average MAE of 6.40° ± 2.12° across all four output channels (bilateral hip sagittal and hip coronal), with per-subject results summarized in Table 5. Among the 11 participants, nine achieved an average MAE below 8°, demonstrating that the LSTM model generalizes well across individuals without subject-specific tuning. The largest per-subject variability was observed for S06, whose hip sagittal errors exceeded those of other participants, likely reflecting individual differences in gait pattern and body proportions.

## 5. Conclusions

This study proposed a camera–IMU fusion framework for gait analysis based on a minimal sensor configuration using a single RGB camera and bilateral shoe-mounted IMUs. Rather than directly fusing camera-based and IMU-based estimations within a single optimization process, the proposed method adopts a modular joint-level architecture in which each sensor stream is independently refined through DEAS-based biomechanical constraint optimization and LSTM-based temporal correction, followed by temporal alignment and selective integration according to joint and axis characteristics.

Experimental results demonstrated encouraging estimation performance for sagittal-plane lower limb kinematics. The proposed pipeline achieved an average MAE of 7.89° ± 1.82° and RMSE of 10.09° ± 2.08° on the held-out test set across sagittal hip, knee, and ankle angles. Leave-one-subject-out cross-validation of the LSTM correction model further confirmed its generalizability across unseen individuals, yielding an average MAE of 6.40° ± 2.12° across bilateral hip angles. The ankle sagittal angle benefited most from IMU fusion (MAE: 5.14–6.96°, accuracy: 83.9–84.4%) through the kinematic chain relationship fusing camera-derived proximal joint angles with IMU-measured foot pitch, confirming the rationale for the joint-level integration strategy. These findings suggest that the foot-level motion information from IMUs and the whole-body pose information from the camera were effectively utilized in a complementary manner.

Another contribution of this study is the LSTM-based refinement of hip joint angles using the well-observed knee angle as a reference input, effectively reducing systematic errors in camera-based hip estimation by exploiting the biomechanical coupling between knee and hip motion during gait, as demonstrated in the ablation analysis.

However, this study has several limitations. The validation was conducted with only 11 healthy participants in a controlled laboratory environment. While LOSO cross-validation provides subject-independent evaluation, the small sample size and the absence of pathological gait data (e.g., Parkinson’s disease, stroke) limit the generalizability of the reported performance to clinical populations and real-world settings. Additionally, the quantitative evaluation is restricted to sagittal-plane joint angles, as non-sagittal components showed high inter-subject variability in the reference measurements and could not be reliably validated. While hip coronal and transverse rotations and ankle inversion/eversion were estimated using complementary filtering, IMU yaw dynamics, and roll-based kinematic chain, respectively, their accuracy remained limited compared to sagittal plane results: cumulative IMU yaw drift limits hip transverse estimation without magnetometer correction, and the small angular range of ankle inversion/eversion (~8–12° during normal gait) makes reliable estimation challenging with the current sensor configuration. Furthermore, the shoe-mounted IMU measures shoe orientation rather than anatomical foot orientation, which may also affect ankle angle estimates.

Future work will extend the proposed method to more complex walking conditions, such as turning and variable-speed walking, and validate its clinical utility using pathological gait data from patients with Parkinson’s disease or stroke. In addition, we plan to incorporate magnetometer-aided yaw estimation and further improve the camera-derived shank tilt compensation to enhance the accuracy of hip transverse and ankle coronal angle estimation, expanding the coverage toward full 3D lower limb kinematics. Furthermore, we aim to explore data augmentation techniques to expand the limited training dataset and improve model generalizability across diverse populations. While recent work has demonstrated the effectiveness of generative data augmentation for scalar gait feature classification [34], extending such approaches to continuous time-series biomechanical waveforms remains an open challenge that we plan to investigate. We also aim to develop the system into a practical gait analysis tool through drift correction for prolonged measurements and real-time feedback.

## Figures and Tables

**Table 1:** Comparison of the representative studies in camera-based, IMU-based, and camera–IMU fusion approaches for lower limb gait analysis.

Approach	Study	Sensor Configuration	Target Joints	Main Limitation
Vision-based	Kanko et al. (2021) [9]	Multiple digital cameras	Hip, Knee	Acceptable proximal; not validated at ankle
Vision-based	Needham et al. (2021) [10]	Multi-camera	Hip, Knee, Ankle	Increased distal joint error
Vision-based	Stenum et al. (2021) [15]	Single RGB camera	Lower limb	Limited ankle kinematics accuracy
Vision-based	Kim et al. (2023) [26]	Single RGB camera	Lower limb	Persistent ankle estimation error
IMU-based	Seel et al. (2014) [16]	Shank + Foot IMUs	Knee, Ankle	Drift; no global pose consistency
IMU-based	Huang et al. (2018) [23]	6 sparse IMUs	Full body	Under-constrained; no visual context
IMU-based	Wang et al. (2023) [27]	3 IMUs (thigh, shank, foot)	Hip, Knee, Ankle	Multiple IMUs required per leg
IMU-based	Jocham et al. (2024) [28]	Bilateral foot IMUs	Foot orientation	No full lower limb reconstruction
IMU-based	Xiang et al. (2024) [29]	Foot-mounted IMU	Ankle	Single joint; no kinematic chain
Fusion-based	Von Marcard et al. (2018) [20]	6 IMUs + Camera	Full body	Complex multi-sensor calibration
Fusion-based	Gilbert et al. (2019) [22]	13 IMUs + Multi-camera	Full body	High computational cost; black-box
Fusion-based	Malleson et al. (2020) [21]	Multi-view + IMUs	Full body	Multi-camera setup required
Fusion-based	Yamamoto et al. (2022) [30]	Single RGB + 1 foot IMU	Lower limb	No temporal correction; single foot
Proposed	This paper	1 RGB camera + 2 shoe-mounted IMUs	Hip, Knee, Ankle	Modular joint-level integration; minimal sensor setup

**Table 2:** Proposed sensor modality and estimation method for lower limb joint angles.

Joint	Plane	Sensor	Estimation Method
Hip	Sagittal	RGB camera	DEAS-based joint estimation, LSTM temporal correction
	Coronal	RGB camera	DEAS-based joint estimation, LSTM temporal correction
	Transversal	Shoe-mounted IMU	IMU yaw angle
Knee	Sagittal	RGB camera	DEAS-based joint estimation
Ankle	Sagittal Coronal	Shoe-mounted IMU	Kinematic chain relationship with fused data

**Table 3:** Characteristics of the participants.

Parameter	Total (n = 11)	Male (n = 7)	Female (n = 4)
Age (years)	62.3 ± 13.8	61.3 ± 10.2	66.3 ± 20.0
Height (cm)	166.2 ± 11.4	173.4 ± 4.7	152.3 ± 6.1
Mass (kg)	70.3 ± 16.5	81.2 ± 11.1	52.7 ± 4.3

**Table 4:** Ablation comparison of DEAS-only and the proposed pipeline on the held-out test set.

Joint	Plane	DEAS-Only		Proposed		Accuracy (%)
		MAE (°)	RMSE (°)	MAE (°)	RMSE (°)	
L Hip	Sagittal	19.00 ± 5.33	22.67 ± 5.86	7.31 ± 1.57	8.96 ± 1.79	86.8
R Hip	Sagittal	18.52 ± 3.99	22.21 ± 4.54	8.87 ± 1.82	10.61 ± 1.98	83.9
L Knee	Sagittal	23.43 ± 6.80	31.00 ± 8.32	9.64 ± 3.16	12.72 ± 4.58	87.0
R Knee	Sagittal	20.09 ± 4.07	27.40 ± 4.88	9.40 ± 2.63	12.60 ± 4.14	87.0
L Ankle	Sagittal	9.00 ± 2.21	10.81 ± 2.30	5.14 ± 0.86	6.36 ± 1.05	84.4
R Ankle	Sagittal	8.36 ± 1.80	10.10 ± 2.06	6.96 ± 1.33	9.28 ± 1.71	83.9
Average		16.40 ± 4.03	20.70 ± 4.66	7.89 ± 1.82	10.09 ± 2.08	85.5

**Table 5:** Per-subject LOSO cross-validation results for the LSTM correction model.

Subject	LHip Sag (°)	RHip Sag (°)	LHip Cor (°)	RHip Cor (°)	Average (°)
S01	6.49	6.07	4.58	3.97	5.28
S02	6.56	7.35	2.91	1.95	4.69
S03	8.44	9.94	3.06	1.23	5.67
S04	10.90	8.56	4.37	2.88	6.68
S05	3.90	4.37	2.00	2.88	3.29
S06	17.86	17.69	4.82	3.70	11.02
S07	8.53	7.25	9.44	2.33	6.89
S08	7.32	6.51	2.61	3.80	5.06
S09	7.70	11.54	2.95	6.81	7.25
S10	5.68	5.20	7.38	2.26	5.13
S11	12.09	18.54	4.06	3.10	9.45
Mean ± Std	8.68 ± 3.63	9.37 ± 4.56	4.38 ± 2.13	3.17 ± 1.40	6.40 ± 2.12

## Data Availability

The data supporting the findings of this study are available from the corresponding author upon reasonable request.

## References

[1] Whittle M.W. (2014). Gait Analysis: An Introduction.

[2] Baker R. (2013). Measuring Walking: A Handbook of Clinical Gait Analysis.

[3] Perry J., Burnfield J.M. (2010). Gait Analysis: Normal and Pathological Function.

[4] Muro-de-la-Herran A., Garcia-Zapirain B., Mendez-Zorrilla A. (2014). Gait Analysis Methods: An Overview of Wearable and Non-Wearable Systems, Highlighting Clinical Applications. Sensors.

[5] Simon S.R. (2004). Quantification of Human Motion: Gait Analysis—Benefits and Limitations to Its Application to Clinical Problems. J. Biomech..

[6] Cappozzo A., Dellacroce U., Leardini A., Chiari L. (2005). Human movement analysis using stereophotogrammetry: Part 1: Theoretical background. Gait Posture.

[7] Baker R. (2006). Gait Analysis Methods in Rehabilitation. J. Neuroeng. Rehabil..

[8] Colyer S.L., Evans M., Cosker D.P., Salo A.I.T. (2018). A Review of the Evolution of Vision-Based Motion Analysis and the Integration of Advanced Computer Vision Methods Towards Developing a Markerless System. Sports Med.-Open.

[9] Kanko R.M., Laende E.K., Davis E.M., Selbie W.S., Deluzio K.J. (2021). Concurrent Assessment of Gait Kinematics Using Marker-Based and Markerless Motion Capture. J. Biomech..

[10] Needham L., Evans M., Cosker D.P., Wade L., McGuigan P.M., Bilzon J.L., Colyer S.L. (2021). The Accuracy of Several Pose Estimation Methods for 3D Joint Centre Localisation. Sci. Rep..

[11] Bazarevsky V., Grishchenko I., Raveendran K., Zhu T., Zhang F., Grundmann M. (2020). BlazePose: On-Device Real-Time Body Pose Tracking. arXiv.

[12] Li J., Xu C., Chen Z., Bian S., Yang L., Lu C. (2022). HybrIK: A Hybrid Analytical-Neural Inverse Kinematics Solution for 3D Human Pose and Shape Estimation. Proceedings of the IEEE/CVF Conference on Computer Vision and Pattern Recognition (CVPR).

[13] Xu Y., Zhang J., Zhang Q., Tao D. (2022). ViTPose: Simple Vision Transformer Baselines for Human Pose Estimation. Advances in Neural Information Processing Systems 35.

[14] Viswakumar A., Rajagopalan V., Ray T., Parimi C. (2019). Human Gait Analysis Using OpenPose. Proceedings of the 2019 Fifth International Conference on Image Information Processing (ICIIP).

[15] Stenum J., Rossi C., Roemmich R.T. (2021). Two-Dimensional Video-Based Analysis of Human Gait Using Pose Estimation. PLoS Comput. Biol..

[16] Seel T., Raisch J., Schauer T. (2014). IMU-Based Joint Angle Measurement for Gait Analysis. Sensors.

[17] Mariani B., Jiménez M.C., Vingerhoets F.J.G., Aminian K. (2013). On-Shoe Wearable Sensors for Gait and Turning Assessment of Patients With Parkinson’s Disease. IEEE Trans. Biomed. Eng..

[18] Washabaugh E.P., Kalyanaraman T., Adamczyk P.G., Claflin E.S., Krishnan C. (2017). Validity and Repeatability of Inertial Measurement Units for Measuring Gait Parameters. Gait Posture.

[19] Picerno P. (2017). 25 Years of Lower Limb Joint Kinematics by Using Inertial and Magnetic Sensors: A Review of Methodological Approaches. Gait Posture.

[20] Von Marcard T., Henschel R., Black M.J., Rosenhahn B., Pons-Moll G., Ferrari V., Hebert M., Sminchisescu C., Weiss Y. (2018). Recovering Accurate 3D Human Pose in the Wild Using IMUs and a Moving Camera. Computer Vision—ECCV 2018.

[21] Malleson C., Collomosse J., Hilton A. (2020). Real-Time Multi-Person Motion Capture from Multi-View Video and IMUs. Int. J. Comput. Vis..

[22] Gilbert A., Trumble M., Malleson C., Hilton A., Collomosse J. (2019). Fusing Visual and Inertial Sensors with Semantics for 3D Human Pose Estimation. Int. J. Comput. Vis..

[23] Huang Y., Kaufmann M., Aksan E., Black M.J., Hilliges O., Pons-Moll G. (2018). Deep Inertial Poser: Learning to Reconstruct Human Pose from Sparse Inertial Measurements in Real Time. ACM Trans. Graph..

[24] Kim J. (2006). On the Global Convergence of Univariate-Dynamic Encoding Algorithm for Searches (uDEAS). Proceedings of the 2006 SICE-ICASE International Joint Conference.

[25] Ha E., Kim J.-W. (2026). A Correction Method for Monocular Camera-based Gait Joint Angle Estimation Using LSTM With Knee Joint Time-series Information. J. Inst. Control Robot. Syst..

[26] Kim J.-W., Choi J.-Y., Ha E.-J., Choi J.-H. (2023). Human Pose Estimation Using MediaPipe Pose and Optimization Method Based on a Humanoid Model. Appl. Sci..

[27] Wang F., Liang W., Afzal H.M.R., Fan A., Li W., Dai X., Liu S., Hu Y., Li Z., Yang P. (2023). Estimation of Lower Limb Joint Angles and Joint Moments during Different Locomotive Activities Using the Inertial Measurement Units and a Hybrid Deep Learning Model. Sensors.

[28] Jocham A.J., Laidig D., Guggenberger B., Seel T. (2024). Measuring Highly Accurate Foot Position and Angle Trajectories with Foot-Mounted IMUs in Clinical Practice. Gait Posture.

[29] Xiang L., Gu Y., Gao Z., Yu P., Shim V., Wang A., Fernandez J. (2024). Integrating an LSTM Framework for Predicting Ankle Joint Biomechanics during Gait Using Inertial Sensors. Comput. Biol. Med..

[30] Yamamoto M., Shimatani K., Ishige Y., Takemura H. (2022). Verification of Gait Analysis Method Fusing Camera-Based Pose Estimation and an IMU Sensor in Various Gait Conditions. Sci. Rep..

[31] Cao Z., Hidalgo G., Simon T., Wei S.-E., Sheikh Y. (2021). OpenPose: Realtime Multi-Person 2D Pose Estimation Using Part Affinity Fields. IEEE Trans. Pattern Anal. Mach. Intell..

[32] Choi J.-Y., Ha E., Son M., Jeon J.-H., Kim J.-W. (2024). Human Joint Angle Estimation Using Deep Learning-Based Three-Dimensional Human Pose Estimation for Application in a Real Environment. Sensors.

[33] Madgwick S.O.H., Harrison A.J.L., Vaidyanathan R. (2011). Estimation of IMU and MARG Orientation Using a Gradient Descent Algorithm. Proceedings of the 2011 IEEE International Conference on Rehabilitation Robotics.

[34] Trabassi D., Castiglia S.F., Bini F., Marinozzi F., Ajoudani A., Lorenzini M., Chini G., Varrecchia T., Ranavolo A., De Icco R. (2024). Optimizing Rare Disease Gait Classification through Data Balancing and Generative AI: Insights from Hereditary Cerebellar Ataxia. Sensors.

